# Improving the Luminescence Performance of Monolayer MoS_2_ by Doping Multiple Metal Elements with CVT Method

**DOI:** 10.3390/nano13182520

**Published:** 2023-09-08

**Authors:** Bojin Zhao, Zongju Huo, Lujie Li, Hongjun Liu, Zhanggui Hu, Yicheng Wu, Hailong Qiu

**Affiliations:** Tianjin Key Laboratory of Functional Crystal Materials, Institute of Functional Crystal, Tianjin University of Technology, Tianjin 300384, China; zbojin@stud.tjut.edu.cn (B.Z.); huozongju@stud.tjut.edu.cn (Z.H.); lilujie2008@gmail.com (L.L.); hjliu@email.tjut.edu.cn (H.L.); hu@tjut.edu.cn (Z.H.); ycwu@tjut.edu.cn (Y.W.)

**Keywords:** doping, MoS_2_, CVT

## Abstract

Two-dimensional (2D) transition metal dichalcogenides (TMDCs) draw much attention as critical semiconductor materials for 2D, optoelectronic, and spin electronic devices. Although controlled doping of 2D semiconductors can also be used to tune their bandgap and type of carrier and further change their electronic, optical, and catalytic properties, this remains an ongoing challenge. Here, we successfully doped a series of metal elements (including Hf, Zr, Gd, and Dy) into the monolayer MoS_2_ through a single-step chemical vapor transport (CVT), and the atomic embedded structure is confirmed by scanning transmission electron microscope (STEM) with a probe corrector measurement. In addition, the host crystal is well preserved, and no random atomic aggregation is observed. More importantly, adjusting the band structure of MoS_2_ enhanced the fluorescence and the carrier effect. This work provides a growth method for doping non-like elements into 2D MoS_2_ and potentially many other 2D materials to modify their properties.

## 1. Introduction

Two-dimensional (2D) transition metal dichalcogenide (TMDCs) materials are considered to have great potential for application in technologies such as integrated circuits, field-effect transistors (FET), and optoelectronic devices [1,2,3]. In order to expand its application circumstances, doping can be used to regulate the performance of TMDC materials. For instance, doping in TMDCs has the potential to modulate their charge carrier concentration, phase, electronic, and optical band structures, as well as their magnetic properties and may give TMDCs new properties and functionalities for various applications [4,5,6,7,8,9,10]. Moreover, doped MoS_2_ structures can be applied in various technologies such as 2D or 3D, including in photodetectors, transistors, thin-film photovoltaic devices, p-n junctions, non-volatile multi-bit data storage memory, ultrasensitive sensors, and photocatalysts [11].

However, the current doping methods are relatively complex and mainly focus on the mutual doping of like elements (e.g., Mo, W, Re, and Nb) in corresponding materials in TMDC, such as MoS_2_, WSe_2_, ReS_2_, and NbS_2_ [12,13,14,15,16,17]. For example, Suh et al. [18] found that the substitutional doping of MoS_2_ with Nb leads to its structural transformation from the 2H to the 3R phase. Nevertheless, the doping of unlike elements still poses significant challenges. Correspondingly, recent theoretical research has found that elements such as Cr, Mn, Fe, etc., if doped into TMDC materials, will greatly alter the properties of TMDC and endow the materials with unique characteristics, such as dilute magnetism and self-spintronic applications [19,20,21]. However, successful examples are rarely reported. For example, it was reported that Er-doped MoS_2_ thin films were obtained by depositing Er onto Mo-thin films and then vulcanizing them, and their optical properties were improved [22,23]. However, this method is not generalizable and requires strict experimental conditions. The domain size of Er-doped MoS_2_ thin films grown through sulfurization is usually relatively small. This requires a doping method that can have universality and ensures the production of high-quality TMDC materials, opening up ideas for this field.

Here, we use MoS_2_ as the representative material of TMDCs and synthesize a high-quality monolayer MoS_2_ through chemical vapor transport (CVT) in a high vacuum-sealed environment. Based on further improving the process, a one-step CVT method was implemented to achieve the doping of IVB and VB transition metal elements, introducing foreign defects to enrich the electronic energy level structure of MoS_2_ itself and improving the luminescence performance of the monolayer MoS_2_ crystal. On the other hand, the doping of lanthanide elements was also achieved using this method, and fluorescence-enhanced MoS_2_ crystals doped with lanthanide elements were obtained. The band structure of MoS_2_ was changed by using the rich electronic energy level of rare earth elements. Findings showed that the fluorescence of MoS_2_ was markedly enhanced after doping [24,25,26]. Moreover, the carrier lifetime was substantially prolonged by the kinetic test. These works are of great significance for further research on 2D MoS_2_ optoelectronic devices and FET transistors [27].

## 2. Materials and Methods

Sample preparation: The Zr, Hf, Gd, and Dy-doped MoS_2_ monolayers were synthesized through the CVT process. Raw materials including MoO_3_ (Alfa Aesar, 99.7%, ≈2.3 mg), ZrO_2_ (Alfa Aesar, 99.99%, ≈0.60 mg), HfO_2_ (Alfa Aesar, 99.99%, ≈1.01 mg), GdCl_3_ (Alfa Aesar, 99.99%, ≈1.22 mg), DyCl_3_ (Alfa Aesar, 99.99%, ≈1.25 mg), S (Alfa Aesar, 99.7%, ≈0.40 mg), and the transport agent of iodine (Alfa Aesar, 99.7%, ≈3.00 mg) powers were mixed evenly and then loaded into one end of a quartz tube. The size of the quartz tube used is 10 mm in inner diameter and 11 cm in length. The mica substrate and raw materials are placed at both ends of the quartz tube and separated by the necking of the quartz tube. Afterwards, the quartz tube is vacuumed to 3–5 × 10^−4^ Pa and sealed. The experiment adopts a single temperature zone tube furnace, with a high temperature zone in the middle and low temperature zone at both ends. The raw material area in the sealed quartz tube is placed in the high-temperature zone, and the substrate area (growth zone) is placed in the low-temperature zone. Set the program to heat at a rate of 40 °C/min to 850 °C, maintain this temperature for 0.5–2 h, and then cool with air to room temperature.

Transfer method: Use a homogenizer to adsorb the doped monolayer MoS_2_ film and rotate it at 3000 rpm for 20 s while dripping PMMA solution onto the surface of the film. Then, remove the sample and place it on a heating plate to heat at 80 °C for 8–10 min to cure. After cooling the sample to room temperature, the PMMA-deposited MoS_2_ film and substrate are separated by soaking in deionized water. Finally, the thin film is transferred to the desired substrate and annealed at 70 °C for 2 h to firmly adhere to the substrate. Then, the PMMA film is washed or soaked with acetone to dissolve and release the MoS_2_ film onto the target substrate.

Optical Spectroscopy measurement: Commercial Confocal microscopy (WITec Alfa-300, Ulm, Germany, OXFORD Instruments) is used to test the PL and Raman data. The testing conditions are room temperature, a laser wavelength of 532 nm, and a spatial resolution of 2 μm. The microscope operates in reflection mode and uses an electrically cooled charge-coupled device camera to detect signals.

The transient reflection (TR) spectrum was obtained through testing using self-built ultra-fast pump–probe spectroscopy. Connect the self-made microscope to the system to achieve micron level resolution. Use a Yb:KGW laser with a working wavelength of 1030 nm (pulse width of 120 fs; a repetition rate of 100 kHz; Pharos, Light Conversion Ltd., Keramiku st. 2B, Vilnius, Lithuania) as the light source. During the testing process, the laser output beam is divided into pump light and probe light. Pump light is used to generate pump pulses (315–2600 nm) to pump the sample. The probe light passes through the delay line and focuses on the YAG crystal, generating continuous pulses to detect the dynamics after pumping. After filtering the pump pulls using a long-wave filter, the reflection spectrum is collected using a complementary metal oxide semiconductor detector. The TR signal is calculated as ΔR/R = (R_pump-on_ − R_pump-off_)/R_pump-off_, where R_pump-on_ and R_pump-off_ are reflection detection signals.

Characterization: This experiment used Olympus BX51M to take and observe optical micrographs of Hf, Zr, Dy, and Gd-doped MoS_2_. In addition, the experiment used Thermo Scientific ESCALAB250Xi (Thermo Scientific, China) to collect XPS data, and all peaks were calibrated with the C1s peak binding energy of foreign carbon at 284.8 eV as the standard. The STEM images of doped-MoS_2_ were obtained using a Titan60-300 (FEI Company, America) at 200 kV.

## 3. Results and Discussion

**Figure 1:**
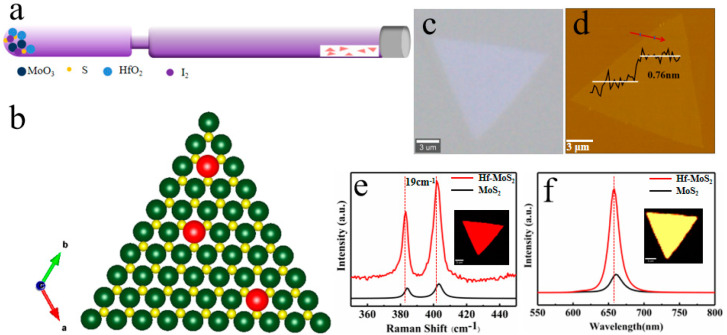
The principle and optical characterization of the Hf-doped MoS_2_ monolayer synthesized by CVT method. (**a**) Simulation diagram of the Hf-doped monolayer MoS_2_ grown by CVT technology. (**b**) Top view of the Hf-doped MoS_2_ atomic model. (**c**) Microscopic optical photo of the Hf-doped 1L-MoS_2_. (**d**) Schematic atomic model of the monolayer Hf-doped MoS_2,_ which shows a thickness of 0.76 nm. (**e**,**f**) Raman/PL spectra of the Hf-MoS_2_ and the pure MoS_2_ were obtained with a 532 nm laser. Inset: Spatially resolved Raman/PL intensity mapping at 385 cm^−1^/657 nm for the Hf-MoS_2_ monolayer.

We chose the Hf element as the representative element for the monolayer MoS_2_ crystals doped with transition metal materials, using the CVT method of a sealed quartz tube. The mixture of MoO_3_/HfO_2_/S/I_2_ powers and a mica substrate were placed on both ends of the quartz tube, as shown in Figure 1a. When using the CVT method to obtain ultra-thin MoS_2_, it is essential to use a way to reduce the transport efficiency of the transport agent. The specific implementation method is to reduce the size of the transport channel between the raw material and substrate (as shown in Figure 1a). This design can separate the substrate and raw materials and slow down the transport rate, thereby controlling the film size and thickness [28]. The final obtained model structure of the Hf-doped MoS_2_ is a 2H-MoS_2_ structure, where Hf replaces the Mo atoms (Figure 1b).

Figure 1c,e,f shows the optical micrographs, Raman, and PL spectra of the Hf-doped onelayer (1L)-MoS_2_ crystal, respectively. From the optical photo, it can be seen that the morphology of MoS_2_ after doping remains almost unchanged, maintaining the growth morphology of pure MoS_2_. In addition, Figure 1d shows the two characteristic Raman vibration modes of the E^1^_2g_ and A_1g_ modes related to the out-of-plane vibration. Similar Raman spectra also confirmed that there was no significant change in the lattice after doping, except for the collective red-shift of the Raman peak positons caused by lattice distortion after atomic substitution. The Raman spectrum characterization measured a 19 cm^−1^ difference in the A_1g_ and E^1^_2g_ vibration peak positions of the doped MoS_2_ [29,30], consistent with the Raman peak position difference between the monolayer MoS_2_. At the same time, the atomic force microscopy (AFM) image in Figure 1d shows the flat surface, with a thickness of 0.76 nm. From the Raman mapping in Figure 1e, a high-quality Hf-doped 1L-MoS2 can be observed, where the two characteristic Raman vibration modes E^1^_2g_ and A_1g_ are related to out of plane vibrations. Figure 1f shows that the PL intensity of the Hf-doped monolayer MoS_2_ is stronger than that of pure MoS_2_, which is about five times stronger. Additionally, the PL peak position is slightly blue-shifted due to the lattice distortion and bandgap changes caused by doping. Simultaneously, the luminescence peak of PL also has a narrow half-peak width, indicating the uniformity of the luminescence quality [31,32]. Therefore, we believe that doping the Hf element introduces the electronic energy level of the Hf element, which enriches the electronic structure of MoS_2_.

**Figure 2:**
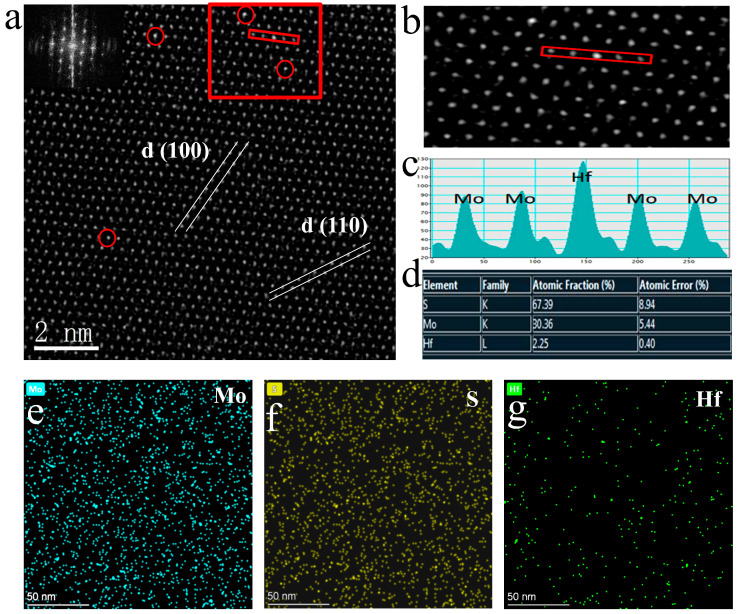
STEM and EDS test results of the Hf-doped monolayer MoS_2_. (**a**) STEM image of the monolayer Hf-doped MoS_2_. Inset: the corresponding FFT-diffraction patterns. (**b**) An enlarged view of the red rectangular area in (**a**). (**c**) The intensity line profile corresponding to the marked area in (**b**). (**d**) The content comparison of Mo, S, and Hf in the Hf-doped monolayer MoS_2_, measured from EDS analysis. (**e**–**g**) Spatially resolved EDS maps collected from the same area in the Hf-MoS_2_ monolayer for the Mo-K, S-K, and Hf-L lines, respectively.

HAAD-STEM can distinguish different atoms according to different brightness, which is caused by different atomic numbers (S (Z = 16), Mo (Z = 42), and Hf (Z = 72)). Based on this, we marked the position occupied by Hf doping in the MoS_2_ monolayer with red circles in Figure 2a. Simultaneously, the plane spacing between two adjacent (100) faces is 0.28 nm, which is consistent with the data of the pure monolayer MoS_2_. However, the measurement found that the distance between two adjacent Mo atoms was 0.32 nm, and compared with pure phase MoS_2_, the distance between faces and atoms increased. This is because the Hf atom is much larger than the Mo atom, leading to slight lattice distortion in MoS_2_.

On the other hand, it proves that the Hf atom has successfully embedded in the lattice of MoS_2_, replacing the position of Mo atoms, which is consistent with HAADF-STEM testing. Furthermore, in Figure 2b, the highlighted area in Figure 2a was enlarged, and a clear distinction between light and dark can be seen. In addition, the illustration in Figure 2a also shows the uniformity and high quality of the thin film material [33].

The profile of the intensity line in Figure 2c corresponds to the selected area in Figure 2b, where Hf and Mo atoms are marked. Except for the bright sites of Hf atoms, all points on the Mo atomic sites exhibit similar contrast, indicating that the Mo atomic vacancies were almost not found on the measured surface [7,34]. Another noteworthy phenomenon is that the strength near the Hf atom substituting the Mo atom is different from that near the Mo atom in other regions (Figure 2c). We supposed that this is due to the generation of new bonding oxygen atoms near the doped Hf atom and the formation of a new O-Hf-S structure by replacing the S atom. Furthermore, EDS spectrum mapping shows that the Hf is evenly distributed among the atoms in the sample, with a doping amount of approximately 1:15 compared to the Mo element (Figure 2d). Moreover, the sum of the ratio of the Mo element to the S element is about 1:2, proving that the Hf element replaces the Mo element. Not only that, the absence of significant lattice distortion and Mo vacancies proves that this CVT method can prepare high-quality monolayer MoS_2_-doped samples [35,36].

**Figure 3:**
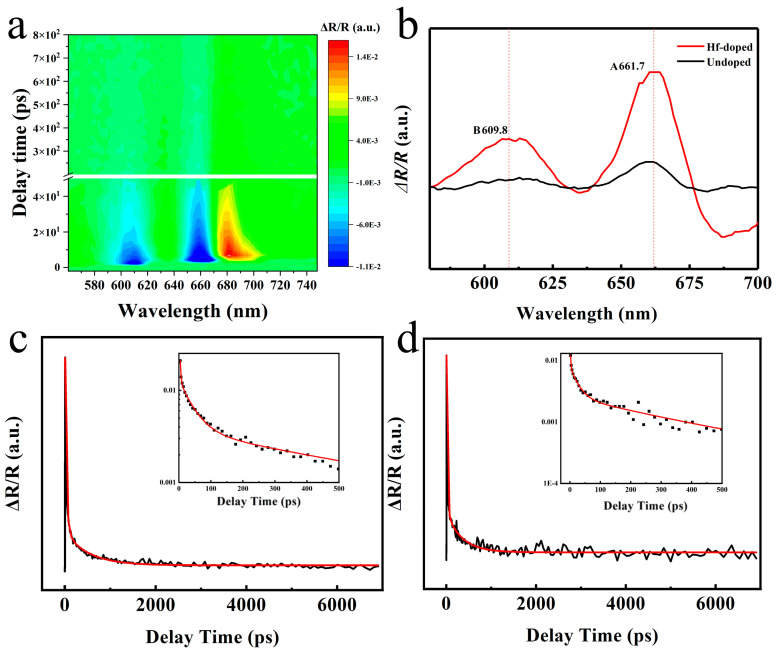
TR dynamics of the Hf-doped. (**a**) TR contour plot of the Hf-doped MoS_2_ 1L monolayer on mica substrate. (**b**) TR spectra extracted from (**a**) with a delay of 4.2 ps under a 532 nm laser excitation. (**c**,**d**) The TR kinetic curves of exciton resonance of A and B obtained through a triexponential fitting were extracted from (**b**). The illustration is divided into an enlarged section, with A and B excitons decaying within 500 ps.

The femtosecond pump–probe is an essential tool for studying the carrier dynamics of Hf-doped MoS_2_ monolayers (please refer to Section 2 for detailed testing information). Firstly, we tested the 2D profile of the transient reflection (TR) spectra of the Hf-doped MoS_2_ monolayer at room temperature with an excitation power of 2 μw under a 530 nm laser. Firstly, we extracted the 4.2 ps time-delayed resonance TR spectrum from Figure 3a, where the two peaks correspond to the A and B excitons of the Hf-doped 1L-MoS_2_ (Figure 3b). In terms of strength comparison, the doped material is much stronger than the pure 1L-MoS_2_, which corresponds to the luminescence enhancement phenomenon in the PL spectrum. Furthermore, the TR kinetic decay curve of the A-exciton resonance of the Hf-doped 1L-MoS_2_ was collected (Figure 3c), and the kinetic decay curve was analyzed through tri-exponential fitting (as shown in Table 1). These fitting results indicate that the relaxation lifetime τ_1_ has increased from 0.91 ps to 2.14 ps after doping, which is believed to be due to the additional defect scattering of hot carriers caused by Hf doping, thereby prolonging the relaxation time. More importantly, the lifetime τ_2_ representing the radiative recombination process has also significantly increased. According to a previous report, the photoluminescence quantum yield (PLQY) enhancement of two different radiation processes can be roughly estimated by the following equation [37,38,39]:

(1) $$\eta =\frac{{\bar{\tau }}_{D}}{{\bar{\tau }}_{P}}$$

Here, ${\bar{\tau }}_{D}$ and ${\bar{\tau }}_{P}$ correspond to the average exciton lifetime of doped and undoped monolayer MoS_2_, respectively. Based on the previous work of our research group [7], we substituted τ_2_ to calculate the PLQY enhancement instead of the average lifetime. The calculated value of η is approximately 1.67, confirming the previously observed phenomenon of PL enhancement after Hf doping. It is worth noting that the calculated value of η based on this formula is an estimated value, far less than the five-times luminescence enhancement obtained through PL testing, which is related to the testing mechanism. TR spectrum is an absorption spectrum collected through a pump probe, while the PL spectrum is based on the spectrum obtained by a streak camera, which provides more accurate data compared to the former. On the other hand, the TR detection system is a platform built by our laboratory, and its collection efficiency is weaker than commercial confocal microscopy detection platforms. Thus, η in PLQY enhancement is underestimated.

It is worth noting that the radiation efficiency is positively correlated with the electron density in the valence band. During the EEA process (τ_3_), exciton scattering causes a non-radiative recombination, transferring energy to another exciton. The additional energy promotes residual excitons to become free charge carriers. Therefore, the increase in carrier density caused by doping is another key factor that enhances PL in addition to PLQY. From Table 1, we can intuitively see that the lifetime τ_3_ of the doped EEA process is much more enhanced compared to the original monolayer MoS_2_, and a longer lifetime promotes radiation recombination and improves PL intensity [40,41,42]. Based on the above discussion, we believe that the enhancement of PL by decay lifetime is crucial.

From the perspective of material structure, combining STEM images and XPS test results (Figure S1, supporting literature), we assume that the doping process introduces doping elements and some oxygen atoms, which can repair vacancies in the monolayer MoS_2_ while suppressing nonradiative recombination, improving carrier lifetime, and enhancing PL. The absence of Mo and S vacancies in the STEM test graphics in Figure 2a–c is also substantial proof. Moreover, the O-Hf-S unit and Hf atoms mentioned earlier can also upgrade the interaction between 1L-MoS_2_ and mica substrate, stabilizing excitons and leading to longer exciton lifetimes, ultimately achieving enhanced PL luminescence [43].

**Figure 4:**
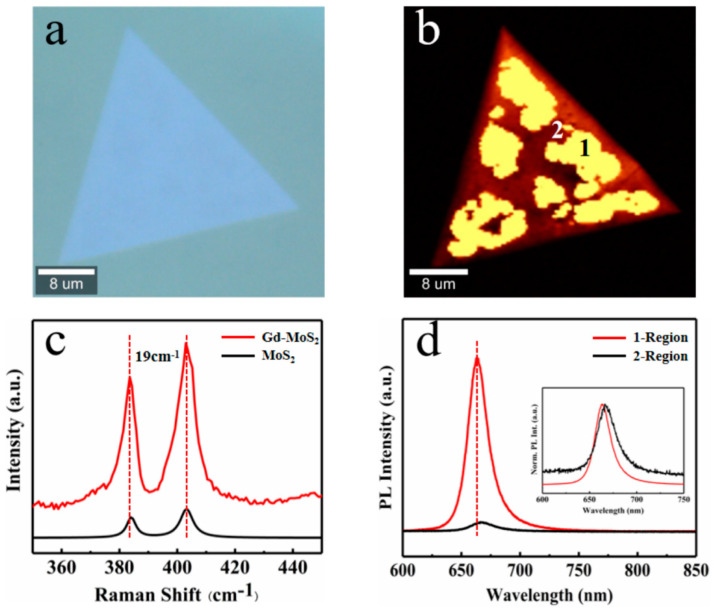
Synthesis of the Gd-doped MoS_2_ monolayer using CVT method. (**a**) A typical optical microscopy image of the Gd-doped triangular MoS_2_ monolayer. (**b**) PL-Mapping spectra of the Gd-doped monolayer MoS_2_. According to luminescence intensity, the spectrum is divided into two regions: bright (1) and dark (2). (**c**) Raman spectra of Gd-MoS_2_ and pure MoS_2_ were obtained with a 532 nm laser. The distance between two vibration peaks is 19 cm^−1^. (**d**) The PL spectra of Gd-doped and undoped 1L-MoS_2_. Inset shows normalized spectra.

Unlike the transition group elements, the sizeable atomic radius of rare earth elements will cause more significant lattice distortion of monolayer MoS_2_ crystals after doping. On this basis, high-quality lanthanide (Gd, Dy)-doped monolayer MoS_2_ crystals were obtained through a one-step CVT process [44,45,46]. Among them, the atomic radius of rare earth elements is large and difficult to dope. The content of rare earth elements in the system is increased by using chlorides with low melting points to improve the success rate of doping. When the amount of chloride increases, it will cause the doped MoS_2_ film to grow thinner and smaller in size. (As shown in Figure S4). Here, we take the Gd-doped monolayer MoS_2_ as an example. Figure 4a presents the optical microscope image of a typical Gd-MoS_2_ monolayer. It exhibits a well-defined triangular shape without grain boundaries, indicating the high quality of the as-grown Gd-MoS_2_ monolayer. (The microscope image of the Dy-MoS_2_ monolayer in Figure S5 of the Supporting Information). The vibration peak position of Figure 4c is almost consistent with that of undoped MoS_2_, indicating that the doped ions have little effect on the lattice of the material, and the distance between the two vibration peaks is 19 cm^−1^, which also indicates that a monolayer Gd-doped MoS_2_ has been prepared. Simultaneously, the Raman mapping in the illustration also shows the high-quality crystallinity of the material.

We analyzed and characterized the PL performance of the monolayer MoS_2_ before and after Gd doping using a Raman spectrometer. It was found that the fluorescent luminescence of Gd-doped MoS_2_ is not uniform (Figure 4b). In the PL mapping of the monolayer MoS_2_, there is a difference of more than 10 times in the luminescence intensity of the two PL regions, bright region 1 and dark region 2. The luminescence intensity of the second region is consistent with the luminescence peak position and intensity of the undoped MoS_2_. This is due to uneven doping, where the doped Gd element affects the lattice of molybdenum sulfide, causing lattice distortion within a certain range, resulting in the uneven PL luminescence of the Gd-doped 1L-MoS_2_ [47,48].

**Figure 5:**
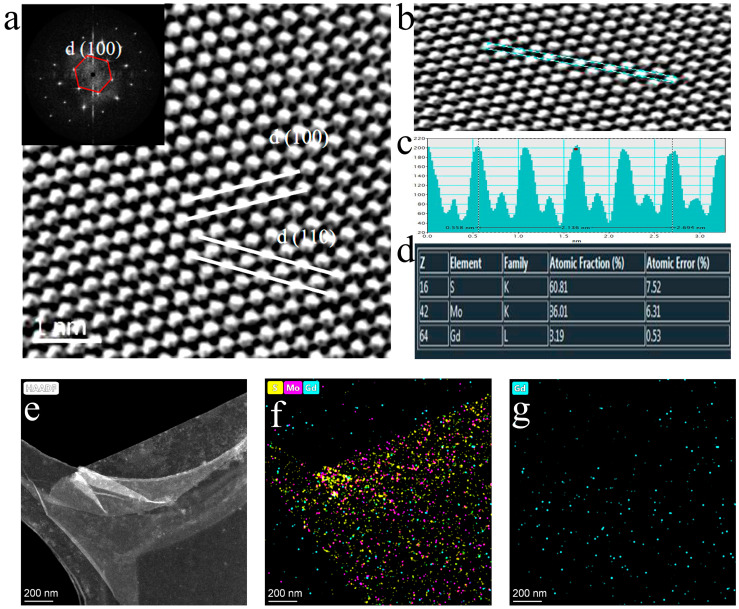
STEM and EDS test results of the Gd-doped monolayer MoS_2_. (**a**) Transmission electron microscope with a probe corrector (STEM) imaging. Scale bar: 1 nm. Inset: the corresponding FFT-diffraction patterns. (**b**) An enlarged view of the red rectangular area in (**a**). (**c**) The intensity line profile corresponding to the marked area in (**b**). (**d**) The content comparison of Mo, S, and Gd in the Gd-doped monolayer MoS_2_, measured from EDS analysis. (**e**) TEM-HAADF images of the Gd-doped monolayer MoS_2_. (**f**,**g**) Spatially resolved EDS maps collected from the same area in a Hf-MoS_2_ monolayer for the Mo-K, S-K, and Gd-L lines.

However, due to the resolution of HR-TEM, the difference between Gd atoms and Mo cannot be diffracted in the STEM images (Figure 5a). On the other hand, diffraction patterns indicate that the doped MoS_2_ is a single crystal with high crystallinity, and the (100) plane spacing is about 0.271 nm, with a distance between two Mo atoms of about 0.316 nm. Compared to the plane spacing and atomic spacing of pure MoS_2_, this confirms the lattice distortion phenomenon caused by doping large Gd atoms into the MoS_2_ lattice. Figure 5e,f, and g show the elemental analysis results of the EDS spectrum, where the Gd is uniformly distributed in the 2D MoS_2_ crystal. The quantitative test results indicate that the doping ratio of Gd is about 3.19% (Figure 5d). In addition, the XPS test results (Supporting Information, Figure S2) also confirmed the successful doping of Gd^3+^ elements.

**Figure 6:**
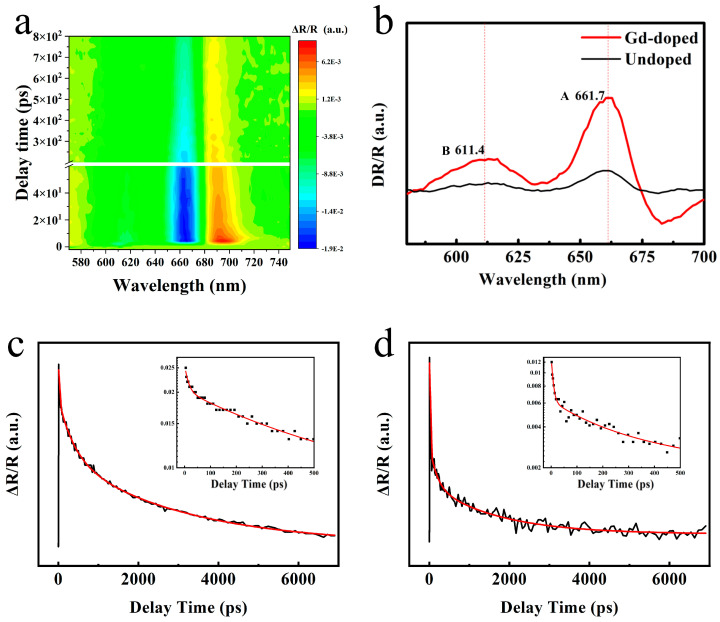
Time-resolved dynamics of the Gd-doped. (**a**) TR contour plot of the Gd-doped MoS_2_ monolayer on a mica substrate. (**b**) TR spectra extracted from (**a**) with a delay of 6.83 ps under a 532 nm laser excitation. (**c**,**d**) The TR kinetic curves of exciton resonance of A and B obtained through a triexponential fitting were extracted from (**b**). The illustration is divided into an enlarged section with A and B excitons decaying within 500 ps.

Furthermore, in order to investigate the reasons for the enhancement of fluorescence intensity after Gd doping and the changing behavior of exciton transitions, transient absorption spectra and exciton lifetime tests were conducted on the sample using a pump detection system to analyze the luminescence kinetics of the sample (Listed in Table 2). As shown in Figure 6b, we found that the absorption peaks of A and B excitons in MoS_2_ doped with Gd shifted red by about 2 nm, and the intensity was significantly enhanced, consistent with the luminescence intensity and peak position changes of the PL spectrum. Figure 6c,d shows that the lifetimes of excitons A and B are much longer than those of pure MoS_2_, corresponding to the significant enhancement of PL strength after Gd doping. The defect capture process in various stages of the exciton lifetime τ_1_ has also significantly increased in length, due to the introduction of more defects after doping and more electrons in the rare earth elements. Exciton recombination process τ_2_ and exciton annihilation process τ_3_. Compared to before doping, the intensity of the PL increased by many times, corresponding to a significant increase in PL intensity. However, due to the large size of rare earth elements, more defects are introduced after doping, resulting in poor crystal quality and lower PL intensity to Ti doping (our research group has previously reported the relevant work [7]).

In addition, we also selected Ti [7], Zr as the IVB group element (Figure S3, Supporting Information), and Dy as the rare earth element for the monolayer MoS_2_ doping experiments. It was found that the doping of the Zr element resulted in a two-fold decrease in PL performance. Tests showed that doping with electrons of different orbital energy levels affected their internal electronic transition behavior and enhanced their luminescence performance. Zr atoms had the same electronic energy levels as Mo atoms, with no transition behavior energy levels. After doping, only the effect of crystal quality deterioration resulted in a decrease in their luminescence performance [49,50,51]. As for the other rare earth element Dy, the doping effect is similar to that of Gd, but the luminescence effect of the Dy-doped monolayer MoS_2_ is only three times enhanced. The data can be found in the supporting literature of Figure S5.

## 4. Conclusions

In summary, we achieved the doping of non-like elements in a monolayer MoS_2_ (including transition group elements Hf, Zr and lanthanide elements Gd, Dy) through a universal CVT method. The doping behavior was demonstrated through testing methods such as Raman, STEM, and XPS. Carrier dynamics tests also indicate that some doped elements entering the host lattice can lead to the additional defect scattering of hot carriers, prolonging relaxation time and radiative recombination processes. On the other hand, the introduced O-X-S (X = Hf, Gd) unit promotes the radiative recombination process and interacts with the substrate. The combined effect of the two enhances the luminescence of PL. This provides reference and guidance for the study of the doping modification of TMDC materials.

## Figures and Tables

**Table 1:** Tri-exponentially fitted lifetime results of A-exciton resonance for the Hf-doped and undoped monolayer MoS_2_.

Exciton	Monolayer	τ_1_ (ps)	τ_2_ (ps)	τ_3_ (ps)
A	Pristine	0.91	24.11	390.08
	Doped	2.14	40.18	522.78

**Table 2:** Tri-exponentially fitted lifetimes results of A-exciton resonance for the Gd-doped and undoped monolayer MoS_2_.

Exciton	Monolayer	τ_1_ (ps)	τ_2_ (ps)	τ_3_ (ps)
A	Pristine	0.91	24.11	390.08
	Doped	14.3	384.28	3012.01

## Data Availability

Data is contained within the article or supplementary material.

## Supplementary Materials

The following supporting information can be downloaded at: https://www.mdpi.com/article/10.3390/nano13182520/s1, Figure S1: XPS measurements on the Zr-/Hf-doped MoS_2_ monolayer; Figure S2: XPS measurements on the Gd/Dy-doped MoS_2_ monolaye; Figure S3: Microscopy image and PL spectra of Zr-MoS_2_; Figure S4: Gd-MoS_2_ film obtained when GdCl_3_ is used as dopant; Figure S5: Microscopy image and PL spectra of Dy-MoS_2_.
