# Supplementary material for: Improving the Luminescence Performance of Monolayer MoS2 by Doping Multiple Metal Elements with CVT Method
Source: Nanomaterials (Basel). 2023 Sep 8;13(18):2520. doi: 10.3390/nano13182520 (PMC10535582; doi:10.3390/nano13182520)
Supplement: Supplementary file 1 [file nanomaterials-13-02520-s001.zip › nanomaterials-2547623-SI.pdf]

nanomaterials

MDPI

Supplementary Materials

Improving the Luminescence Performance of Monolayer MoS_2 by Doping Multiple Metal Elements with CVT Method

Bojin Zhao, Zongju Huo, Lujie Li, Hongjun Liu, Zhanggui Hu and Yicheng Wu and Hailong Qiu *

Tianjin Key Laboratory of Functional Crystal Materials, Institute of Functional Crystal, Tianjin University of Technology, Tianjin 300384, China; zbojin@stud.tjut.edu.cn (B.Z.); huozongju@stud.tjut.edu.cn (Z.H.); lilujie2008@gmail.com (L.L.); hjliu@email.tjut.edu.cn (H.L.); hu@tjut.edu.cn (Z.H.); ycwu@tjut.edu.cn (Y.W.)

* Correspondence: qiu@tjut.edu.cn

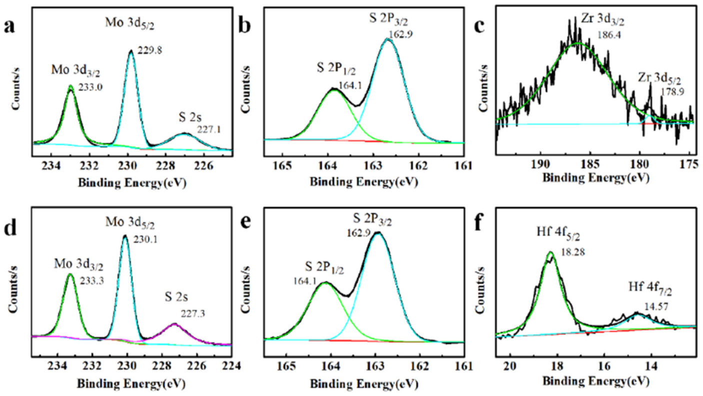

Figure S1. XPS measurements on the Zr−/Hf−doped MoS_2 monolayer. (a)−(c) XPS scans Mo 3d, S 2p, and Zr 3d core levels collected from Zr−doped MoS_2 sample. (d)−(e) XPS scans Mo 3d, S 2p, and Hf 4f core levels collected from Hf−doped MoS_2 sample.

The binding energies of $Mo^{4+}(3d_{3/2})$ and $Mo^{4+}(3d_{5/2})$ orbitals doped with Zr and Hf increased by 0.1 eV and 0.2 eV, respectively, compared to undoped ones. This is due to the small electronegativity of Zr and Hf, which leads to weaker binding on S atoms after replacing Mo atoms, resulting in stronger interaction between Mo and S atoms. It also affects the bonding mode of Mo atoms, which indirectly proves the formation of O-Hf-S bonds. But the size of Zr atoms and Mo atoms is similar, causing less deformation, so the enhancement effect is not as significant as that of Hf element. In addition, we also measured the binding energies of $Zr^{4+}(3d_{3/2})$, $Zr^{4+}(3d_{5/2})$, and $Hf^{4+}(4f_{5/2})$, $Hf^{4+}(4f_{7/2})$ orbitals, verifying the successful doping of Zr and Hf elements.

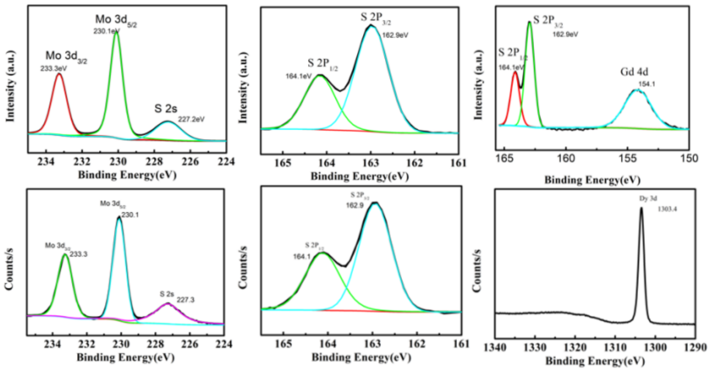

Figure S2. XPS measurements on the Gd−/Dy−doped MoS_2 monolayer. (a)−(c) XPS scans Mo 3d, S 2p, and Gd 4d core levels collected from Gd−doped MoS_2 sample. (d)−(e) XPS scans Mo 3d, S 2p, and Dy 3d core levels collected from Dy−doped MoS_2 sample.

Similar to the doping of transition group element Hf, the doping of Gd and Dy also affects the $3d_{3/2}$ and $3d_{5/2}$ orbitals of Mo, and the peak displacement increases by 0.2 eV after doping. The peak position of the 2P orbital of S remains unchanged. Correspondingly, the chemical environment of Gd and Dy itself is also affected by the hexagonal lattice of MoS_2, and their binding energy shifts towards a larger direction. Here, we believe that they are still +3 valence ions.

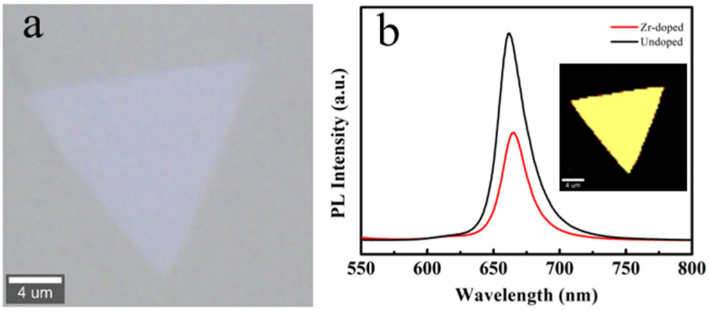

Figure S3. (a) The optical microscopy image of Zr−doped MoS_2 monolayer. The scale bar is 4 μm. (b) PL spectra of Zr−MoS_2 and pure MoS_2 were obtained with a 532 nm laser. Inset: Spatially resolved PL intensity mapping at 661 nm for Zr−MoS_2 monolayer.

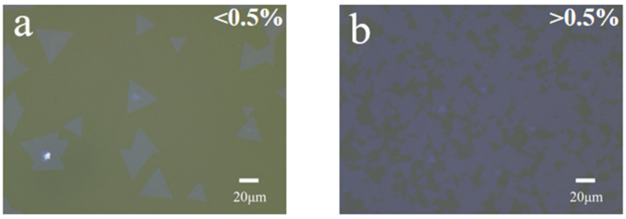

Figure S4. Gd−MoS_2 film obtained when $GdCl_3$ is used as dopant.(a) and (b) are the optical microscopy image of Gd−MoS_2 films when the $GdCl_3$ content is less than 0.5% and more than 0.5%, respectively.

Due to the low melting and boiling points of chlorides and their ability to transport, when the amount of $GdCl_3$ added is low, the grown samples are mostly isolated triangles with a size of 20 μm (Figure S4a); When the amount of $GdCl_3$ added increases, $GdCl_3$ also plays a transport role, leading to more nucleation on the growth substrate, large area film formation, and only a few very small triangles (Figure S4b). Therefore, the addition amount should be minimized as much as possible to avoid significantly affecting the effective material partial pressure in the system and affecting the synthesis of single−layer MoS_2 crystals.

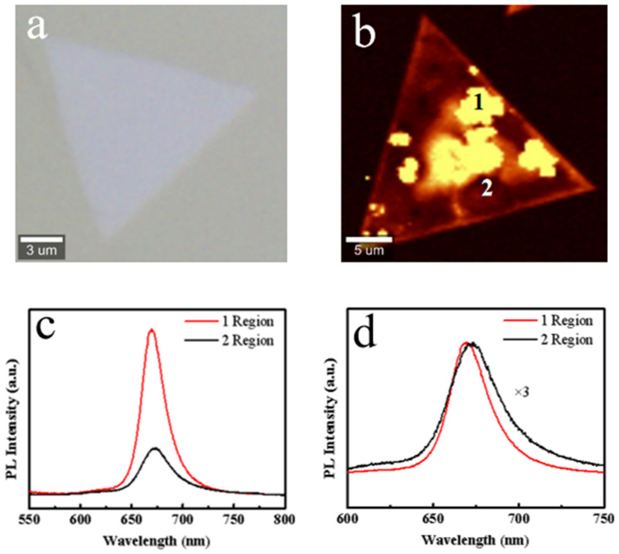

Figure S5. (a) The optical microscopy image of Dy−doped MoS_2 monolayer. (b) PL−Mapping spectra of Dy−doped monolayer MoS_2. According to luminescence intensity, the spectrumof (b) is divided into two regions: bright (1) and dark (2). (c) The PL spectra of Dy−doped and undoped MoS_2 monolayer and (d) shows a three fold difference in intensity between the two regions after normalization.
